# Editorial: Using Genomics, Metagenomics and Other “Omics” to Assess Valuable Microbial Ecosystem Services and Novel Biotechnological Applications

**DOI:** 10.3389/fmicb.2019.00151

**Published:** 2019-02-12

**Authors:** Diana E. Marco, Florence Abram

**Affiliations:** ^1^Faculty of Exact, Physical and Biological Sciences, CONICET, Córdoba National University, Córdoba, Argentina; ^2^Functional Environmental Microbiology, School of Natural Sciences, National University of Ireland Galway, Galway, Ireland

**Keywords:** genomics, metagenomics, meta-omics, microbial ecology, ecosystem services, biotechnology, industrial processes, natural products

Most ecosystem services [defined as the benefits people obtain from ecosystems, Millennium Ecosystem Assessment (2005)] and goods human populations rely on are provided by microbial populations and communities. Indeed, numerous provisioning services (e.g., food and enzymes for industrial processes); regulating services (e.g., water quality, contamination alleviation, and biological processes such as plant-microbial symbioses); and supporting services (e.g., nutrient cycling, agricultural production, and biodiversity), are mediated by microbes. Thus, to preserve and protect these ecosystems, currently facing climate change and anthropologic activities-related challenges, as well as to harness some of the naturally microbial occurring processes, a deep understanding of the microbiology underpinning ecosystem functioning is required. To this end, experimental strategies deploying metagenomics, and other meta-omics technologies have been implemented. The resulting enhanced knowledge directly translates into the emergence of new applications in an unlimited variety of areas across all microbial ecosystem services and goods. To compile an updated overview of these new developments, we called for contributions to this Research Topic. Our expectations were amply rewarded by a high number of submitted manuscripts, for which we are greatly indebted to the authors.

We grouped the corresponding articles under different research themes as follows: (i) development of innovative industrial processes, (ii) discovery of novel natural products, (iii) advancement of new agricultural methods, (iv) amelioration of negative effects of productive or natural microbiological processes, (v) food security and human health, (vi) archeological conservation, and (vii) methodological developments. Some theme overlap is naturally anticipated due to the complex nature of the microbial processes investigated. For example, the improvement of agricultural practices not only presents an economic significance but also may contribute to the amelioration of some pressing environmental issues. The diversity of research showcased in this special issue demonstrates the tremendous potential of omics methods for advancing knowledge and underpinning the development of novel biotechnologies.

Microbial processes have been harnessed by human populations to produce many goods like food and beverages from ancient times. More recently, a variety of industries based on microbial functions began to develop and today microbial-based applications from industrial enzymes to new drugs discovery are widespread (Okafor and Okeke, 2017). The application of omics methods to microbial communities driving milk fermentation for cheese production (Jonnala et al.), flax fiber production (Djemiel et al.), or nutrient acquisition from diet of goat, lamb and piglets (Chen et al.; Lei et al.; Yang et al.), allows for the formulation of new and more efficient production methods. Similarly, optimization avenues for industrial processes of economic and environmental importance can be designed based on knowledge gained from omics investigations.

For example, insights from metatranscriptomics analysis led to the optimisation of biogas production during the anaerobic digestion of microalgae, Córdova et al.; Joyce et al. used metaproteomics in conjunction with 16S rRNA profiling of DNA and cDNA to investigate the anaerobic digestion of perennial grass to produce second-generation biofuels. Microbial groups involved in the anaerobic process were identified, and the functional importance of Clostridia was highlighted by the authors. Wintsche et al. investigated the effects of trace elements depletion during the anaerobic digestion of distillers grains. Using *mcrA* gene amplicon sequencing and metaproteomics, activity shifts within methanogenic communities and the importance of *Methanosarcina* for reactor performance stabilization under critical conditions were highlighted. Tackling the issue of contaminant prevalence in wastewater, González-Martínez et al. investigated the effect of antibiotics exposure in autotrophic nitrogen removal systems for wastewater treatment combining 16S rRNA gene amplicon sequencing and metatranscriptomics.

Omics and meta-omics methodologies also hold great potential for facilitating the screening of secondary microbial metabolites for biotechnological and pharmaceutical industries (Wang et al.; Cuadrat et al.). Using metabolomics to study mutants of *Synechocystis* sp., Shi et al. identified the functions of several novel transcriptional regulators involved in response to diverse environmental stresses (heat, heavy metals), tolerance to ethanol and carbohydrate transport and metabolism. Using comparative genomics, Leyn et al. could unveil a hierarchical carbon flow from cyanobacteria to heterotrophs within benthic microbial-mat derived consortia. The authors reconstructed carbohydrate utilization pathways and identified glycohydrolytic enzymes, carbohydrate transporters and pathway-specific transcriptional regulators in the heterotrophic members of the bacterial consortia. This study revealed novel functional roles of 171 genes, and the utilization capabilities for 40 carbohydrates and their derivatives by the mat, opening the avenue for potential biotechnological applications.

Another important service provided by microbial communities is biofertilization, *via* the synthesis of plant nutrients or phytohormones, the mobilization of soil compounds, the protection of plants under stressful conditions, the defense against plant pathogens (García-Fraile et al., 2015), and biological nitrogen fixation which converts bio-unavailable N_2_ gas to plant-available ammonia (${\text{NH}}_{4}^{+}$) (Bedmar et al., 2013). All these microbial activities allow for a more environmentally friendly agriculture by diminishing the use of chemical fertilizers and toxic compounds. Understanding the structure and functioning of nitrogen-fixing and plant growth promoting microbiomes using omics methods led to improvements in crop management including sugarcane (Li et al.) rice, (Bai et al.; Gu et al.) and maize (Correa-Galeote et al.; Silva et al.), while avoiding or diminishing the use of artificial fertilizers. Using a functional metagenomics approach, Ahmed et al. screened for microbial salt tolerant genes that could be used for producing bioactive compounds to improve crop production under high saline conditions.

Other valuable services provided by microorganisms are environmental bioremediation and amelioration of negative consequences of soil and water contamination from different human activities (Shah et al., 2011). Although many microbes with a bioremediation potential have been isolated and characterized, in most cases a single microorganism cannot completely degrade a given pollutant or be effective in naturally prevalent *in situ* mixed contaminations (Dangi et al., 2018). A variety of pollutants such as arsenic, byphenil, phenanthrene, nitrate, and others can be degraded by microbial consortia, and in this context, the use of omics methodologies, can lead to a better understanding of the mechanisms underlying environmental detoxification. For example, Garrido-Sanz et al. used metagenomics to model the biodegradation of byphenil in contaminated soils and could assign reactions and pathways to specific bacterial groups. A metatrascriptomics study by Liu et al. showed that cooperation among strains (elicited by low soil pH) within microbial consortia improved tetrahydrofuran remediation efficiency compared to single microbial strains activity. Nitrate (${\text{NO}}_{\text{3}}^{-}$) contamination (mainly from agriculture) of soils and freshwater bodies is a major environmental issue, causing many wildlife and human health problems. High ${\text{NO}}_{\text{3}}^{-}$ water concentrations contributes to eutrophication and cause damage to the hemoglobin of aquatic organisms when under its reduced form, nitrite (${\text{NO}}_{2}^{-}$). Drinking water containing high levels of ${\text{NO}}_{\text{3}}^{-}$ and ${\text{NO}}_{2}^{-}$ lead to methemoglobinemia (Greer and Shannon, 2005), while ${\text{NO}}_{\text{3}}^{-}$ can be transformed in the digestive tract in carcinogenic nitrosamines (Craddock and Henderson, 1986). Denitrification is a key microbe mediated process of the nitrogen cycle occurring in low oxygen environments that converts ${\text{NO}}_{\text{3}}^{-}$ to inert nitrogen gas (N_2_) and thus ameliorates the effects of environmental nitrogen pollution. However, most denitrifying bacteria are not able to complete the pathway and emit nitrous oxide (N_2_O) and nitric oxide (NO) as intermediate products. While NO contributes to acid rain, N_2_O is a greenhouse gas with many fold greater potential for global warming compared with that of CO_2_, and a main cause of ozone layer depletion (Bates et al., 2008). Thus, knowing the structure and functioning of denitrifying microbiomes is of paramount importance to device strategies for dealing with nitrate contamination, and the use omics methods is making an important contribution to this end. By using metagenomics to study genes involved in the denitrification pathway of freshwater ponds contaminated with${\text{NO}}_{\text{3}}^{-}$, Chen et al. proposed a strategy for treating wastewater effluents by regulating the C/N ratio through the addition of extra organic carbon, to obtain higher denitrification efficiency. By using metagenomics, metatranscriptomics, and metaproteomics, Lindemann et al. investigated the flow of nitrogen and other elements within phototrophic microbial consortia. Among other interesting results, the authors identified bacterial genomes encoding for nitrate and nitrite reductases needed for denitrification. They also reported that niche partitioning around nitrogen sources may structure the community when microorganisms directly compete for limiting phosphate. Using metagenomics and a phylogenetic placement approach, Fuchsman et al. characterized microbial genes for anoxic N cycling in metagenomes from samples recovered from an oxycline in the Eastern Tropical North Pacific oxygen deficient zone and as such provided an overview of the diverse microbial players driving this process. As previously noted, biofertilization offers an environmentally sustainable option for enhancing crop production. This holds particularly true for legumes, such as soybeans, which establish symbiotic relationships with rhizobia. However, while rhizobia, located in root nodules, fix atmospheric N_2_ for the plants, thus reducing the need for fertilization, they also commonly perform incomplete denitrification. This results in N_2_O production in root nodules and the corresponding emissions are increased during flooding (causing low oxygen conditions) (Tortosa et al., 2015). As an example of this situation, in Argentina, one of the main soybean-producers countries, about 20,000,000 ha were sown with soybean in 2016 (FAOSTAT), of which more than 700,000 ha were flooded (BCBA, 2017) (BCBA Report, 2017). By using the soybean endosymbiont *Bradyrhizobium diazoefficiens* and *in vitro* transcription (IVT) activation assays, Torres et al. were able to dissect the fine regulatory mechanisms involved in the control of the key steps in N_2_O reduction to N_2_ in response to low oxygen. The authors envisage that their findings should help to establish action plans for the development of practical strategies for N_2_O emission mitigation from legume crops. Another important agricultural source of greenhouse gases is livestock production, especially ruminants, that produce methane (CH_4_) a greenhouse gas 23 times more potent than CO_2_ (IPCC, 2014). CH_4_ is a byproduct of microbial feed fermentation inside the rumen, but is also produced by other non-ruminant herbivores like rabbits. Using 16S rRNA gene amplicon sequencing, Mi et al. could correlate microbial community structure differences with lower methane yields in rabbits when compared to sheep. Hydrogen utilization pathways were found to differ between the two animal species. Indeed, the authors reported a lower relative abundance of hydrogen-producing microbes and methanogens in rabbits, as well as an increased abundance of homoacetogens converting hydrogen to acetate.

A recent development in the application of omics technologies is in ecosystem health monitoring for species conservation and wildlife protection (Antwis et al., 2017). Using amplicon-based DNA sequencing of the internal transcribed spacer 1 (ITS1) region, Kirker et al. demonstrated that soil fungal community composition is impacted by long-term exposure to wood preservatives. Corals are among the most endangered species, and their cover and diversity around the world is declining fast. Chimetto Tonon et al. developed a qPCR assay for the monitoring of coral pathogens useful to determine their impact on coral reef ecosystems. In a different approach, Levin et al. focused on *Symbiodinium*, the coral photosymbiont, whose stress-induced loss causes coral bleaching. Using available sequencing data from *Symbiodinium* the authors developed a testable expression construct model that incorporates endogenous *Symbiodinium* promoters, terminators, and genes of interest to enhance the photosymbiont stress tolerance and thus, that of coral reefs.

Multi-omics approaches are also currently deployed to assist risk management in food safety and quality (Cocolin et al., 2017). Using a comparative transcriptomics analysis of *Monascus purpureus* (a yeast used as food colorant that also produces citrinin, a compound with nephrotoxic, hepatotoxic, and carcinogenic activities) and a mutant strain, Liang et al. were able to identify the mechanisms underlying pigment and citrinin biosynthesis. These findings will inform the construction of genetically engineered *Monascus purpureus* strains unable to produce citrinin and optimized for pigment synthesis. *Listeria monocytogenes* is an important food-borne pathogen that causes listeriosis, a dangerous disease with human life compromising consequences. Using transcriptome analysis and sequence alignment, Zhang et al. identified six genes related to D-allose metabolism only present in the genomes of lineage II strains. This finding will benefit isolation strategy and epidemiological research of *L. monocytogenes*.

Recognizing the utmost importance of microbiomes to human health, the Human Microbiome Project (Nelson and White, 2010) was launched in 2007 to provide unprecedented insights into our microbiota. While initially most of the information was derived from 16S rRNA amplicon sequencing and metagenomics, the second phase of the project, launched in 2014, called the Integrative Human Microbiome Project (iHMP), aims to create integrated longitudinal datasets from microbiome and host using multiple omics technologies (https://hmpdacc.org/ihmp/). In that context, Banerjee et al. investigated microbial diversity in four major types of breast cancer using whole genome and transcriptome amplification and a pan-pathogen microarray (PathoChip) strategy. The authors detected unique and common viral, bacterial, fungal, and parasitic signatures for each of the breast cancer types. This information will underpin better prognosis, treatment strategies and clinical outcomes. Liu et al. investigated the microbiome of sputum and oropharyngeal swabs in patients with chronic obstructive pulmonary disease (COPD) using 16S rRNA and ITS amplicon sequencing, and found that the two sample types generated rather similar taxonomic profiles. The finding from this work will contribute to the design of easier methodology for medical sampling in the context of COPD patients.

Omics methodologies have also been recently applied to the field of museum objects and archeological remains preservation. Using a combination of culture-independent and culture-dependent methods Liu et al. investigated the microbial communities responsible for the biodeterioration of antique museum objects and identified fungal and bacterial taxa responsible for the deterioration. The findings will inform the future planning of biocide treatment of museum antiques. In another example, Liu et al. determined the fungal community structure of a wooden tomb from the Western Han Dynasty (206 B.C.−25 A.D.) in China. ITS1 gene amplicon sequencing identified a total of 114 genera distributed across five fungal phyla, with a dominant member, *Hypochnicium* sp. WY- DT1. This fungus was further demonstrated to possess the ability to degrade cellulose and lignin and therefore represents a serious threat to the preservation of wooden archeological remains.

Finally, a set of articles reports on new methodological developments, to deal with omics analysis related challenges. Miyazaki et al. proposed a method to tackle the lack of specificity of 16S rRNA for *Escherichia coli*, a species previously reported as able to harbor foreign 16S rRNA. To circumvent this problem, the authors designed a new primer set for 16S rRNA genes with no overlap with potential mismatch sites formerly detected. Zhong et al. proposed an *in silico* decontamination methodology for the investigation of microorganisms present in ice cores dated 20–30,000 years from the Tibetan Plateau. A series of controls were used to assess contaminant microbial diversity and abundances, which were removed *in silico* from the field samples data. As sequencing methods invariably lead to extensive datasets, more sophisticated and user-friendly methods for data analysis are required. Bhuvaneshwar et al. describe an open source bioinformatics pipeline (viGEN), which allows for the detection and quantification of viral RNA, and variants from viral transcripts. This pipeline can be used to provide novel biological insights into microbial infections and tumorigenesis. Parmar et al. review the use of genomics, transcriptomics, proteomics, and metabolomics methodologies as well as the associated bioinformatics tools to infer phylogenetic affiliation and function of bacteriophages and their impact on diverse microbial communities.

Finally, it is worth highlighting that meta-omics technologies are now commonly used in combination, as each omics provides a different and complimentary level of information on the ecosystem under investigation (Meiring et al., 2011). Indeed several studies from this special issue (e.g., Joyce et al.; González-Martínez et al.; Lindemann et al.) and in recent literature, combine multiple omics to better address microbiome structure and functioning in the context of environmental services, goods, and biotechnological applications. However, multi-omics datasets integration remains challenging and new modeling and statistical tools like multi-layer network theory and artificial intelligence methodologies are being developed to this end (Haas et al., 2017).

We believe that the articles published in this Research Topic provide an updated, high-quality overview of current work in the field. This body of research makes a valuable contribution to the understanding of microbial ecosystem services, and expands the horizon for finding and developing new and more efficient biotechnological applications.

## Author Contributions

DM drafted the manuscript, FA revised the draft and both authors agreed to the final version. DM proposed the Research Topic theme and the articles were edited by DM and FA.

### Conflict of Interest Statement

The authors declare that the research was conducted in the absence of any commercial or financial relationships that could be construed as a potential conflict of interest.
